# Clinical outcome of Gamma Knife radiosurgery (GKRS) for indirect carotid-cavernous fistulas (CCFs)

**DOI:** 10.1097/MD.0000000000047511

**Published:** 2026-02-20

**Authors:** Eun Jeong Koh, Jung-Soo Park

**Affiliations:** a Department of Neurosurgery, Research Institute of Clinical Medicine, Biomedical Research Institute, Jeonbuk National University Hospital, Jeonbuk National University, Jeonju-si, Jeollabuk-do, Republic of Korea.

**Keywords:** carotid-cavernous fistula, Gamma Knife radiosurgery, indirect

## Abstract

Gamma Knife radiosurgery (GKRS) has shown promising results in the treatment of carotid-cavernous fistulas (CCFs); however, it remains widely regarded as an adjuvant modality. This study aims to evaluate the efficacy of GKRS as a standalone treatment for indirect CCFs and to compare our results with those reported in the literature. A retrospective chart review was conducted on 20 patients with indirect CCFs who underwent GKRS between December 2017 and December 2023. The mean follow-up duration was 47.4 months. GKRS was performed using a marginal dose of 16 to 18 Gy at the 50% to 60% isodose line. Clinical symptoms and imaging findings were evaluated at 3, 6, and 12 months after treatment using magnetic resonance imaging and magnetic resonance angiography. Of the 20 patients, 6 were classified as Barrow type B, 10 as type C, and 4 as type D. The mean target volume was 0.53 cm^3^ (range, 0.13–1.64 cm^3^). One patient with bilateral symptoms underwent bilateral GKRS. Four patients with contralateral symptoms were treated at the primary fistula site in 2 patients and the symptom site in 2 patients. Pretreatment symptoms included eyelid edema in 19, elevated intraocular pressure (IOP) in 16 patients (mean IOP, 27.7 mm Hg; range, 19–42 mm Hg), chemosis in 10, exophthalmos in 9, ocular pain in 9, headache in 8, and diplopia in 7 patients. Most symptoms resolved within 3 months (mean 1.3 months), except for elevated IOP, which normalized after about 6 months. The mean time to complete radiologic obliteration was 5.3 months (range, 1–12 months). The overall obliteration rate was 100%, and there was no complication or recurrence. GKRS achieved complete occlusion of indirect CCFs with excellent symptom resolution and no adverse events. These findings support the consideration of GKRS as a definitive, noninvasive treatment option for selected patients with indirect CCFs.

## 1. Introduction

A carotid-cavernous fistula (CCF) is an abnormal communication between the cavernous sinus and the carotid arterial system. CCFs can be classified by cause (traumatic vs spontaneous), velocity of blood flow (high vs low flow), and anatomy (direct vs indirect or dural, internal carotid vs external carotid vs both).^[1,2]^

Indirect carotid-cavernous fistulas (iCCFs) are low-flow pathological shunts between the branches of the internal and/or external carotid artery and the cavernous sinus. The symptoms are revealed differently according to the relationship between adjacent venous tributaries and the cavernous sinus. It can lead to fatal ophthalmologic situations such as blindness associated with severe exophthalmos and secondary glaucoma, cranial nerve paralysis, and cerebral hemorrhage if treatment is not administered at the appropriate time, so CCF is a disease that requires active treatment.^[3]^ It is important to detect the condition early and treat it with less invasive methods before serious symptoms occur to minimize patient discomfort and complications.

Travers did ligation of the internal carotid artery (ICA) for control of CCF in 1811.^[4]^ Since then, many physicians developed various approaches to control CCF with reducing complications. Current management options for iCCFs include microsurgery, endovascular embolization, stereotactic radiosurgery (SRS), and various combinations thereof.^[5,6]^ In 1979, the 1st radiosurgery was performed by Barcia-Salorio.^[7]^ Since then, radiosurgery has been used as a treatment modality in some cases, most often as an adjunctive treatment, in combination with other treatments, or when other treatments have failed.

Gamma Knife radiosurgery (GKRS) is a form of SRS that delivers a highly focused radiation dose to the target lesion with submillimetric precision, thereby minimizing radiation exposure to adjacent structures.^[8]^

Recently, clinical results and meta-analyses have been reported showing that radiosurgery alone can obliterate CCF without adverse effects.^[9–12]^ Long-term follow-up results have been reported that GKRS is a safe treatment that does not raise concerns about the effects of radiation. Therefore, GKRS can be considered as a monotherapy for iCCF patients in situations other than an ophthalmologic emergency. We report the results of standalone GKRS in iCCF patients at our hospital and compare them with the results of previously reported papers.

## 2. Materials and methods

This retrospective study used only de-identified clinical and imaging data without direct patient contact; therefore, Institutional Review Board approval and informed consent were waived.

### 2.1. Patient population

We consecutively enrolled all patients with iCCFs who were treated with GKRS alone at our institution between December 2017 and December 2023. A total of 20 patients met these criteria and were included in the analysis. For each case, we evaluated the time interval from symptom onset to GKRS, classification of CCF type, clinical symptoms, feeding arteries, draining veins, and the duration from GKRS to clinical symptom improvement and radiologic resolution.

### 2.2. Confirm the type of indirect CCF and radiosurgery technique

**Figure 1.**
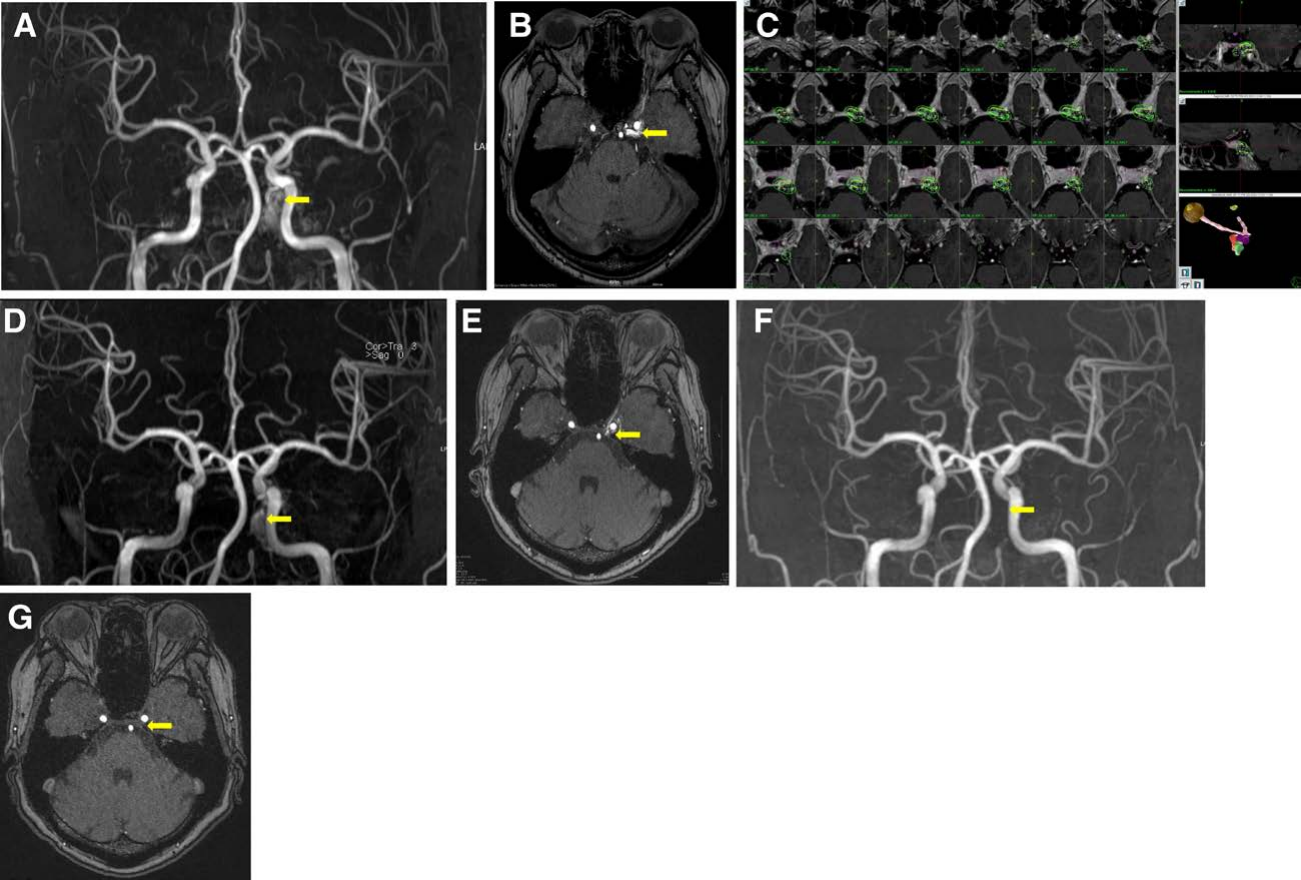
Radiological findings before and after Gamma Knife radiosurgery (GKRS). (A, B) Pre-GKRS magnetic resonance angiography (MRA) and time-of-flight (TOF) source images demonstrate abnormal arteriovenous flow between the internal carotid artery and the cavernous sinus. (C) Radiosurgical treatment plan image. (D and E) Follow-up MRA and TOF source images obtained 6 months after GKRS show a marked reduction in abnormal flow. (F and G) Follow-up MRA and TOF images obtained 12 months after GKRS demonstrate complete obliteration of the abnormal shunt. GKRS = Gamma Knife radiosurgery, MRA = magnetic resonance angiography, TOF = time-of-flight.

We performed transfemoral angiography to confirm the fistula site and check the feeding arteries. We classified indirect CCF using Barrow’s classification.^[1]^ We treated all patients using Leksell Gamma Knife Perfexion (Elekta AB, Stockholm, Sweden) under local anesthesia with stereotactic Leksell G-frame (Elekta AB, Stockholm, Sweden). All patients underwent 1.5 T magnetic resonance imaging (MRI), including time-of-flight (TOF) MRI, Gd-enhanced T1-weighted images, T2-weighted images, and constructive interference in steady state or proton density images (Fig. 1A and B). In the 1st 10 patients, digital subtraction angiography was used to define the CCF target boundary. In the last 10 patients, treatment planning was conducted using magnetic resonance angiography (MRA) TOF images as the primary image without digital subtraction angiography.

We planned radiosurgery on a pre-enhanced magnetic resonance source image of TOF. We delineated the target, which was shown in a simultaneously high signal area with ICA comparing with the normal cavernous sinus area. We restricted the target area to shown early high signal changed region and along the involved cavernous sinus wall. Figure 1C shows the plan image. GKRS was performed with a single fraction with a stereotactic frame, and the irradiated marginal dose to the target was 16 to 18 Gy with 50% to 60% isodose line. Targeted volume, number of isocenters, and conformity index were reviewed. Dose to the optic apparatus and ICA were calculated to avoid complications.

### 2.3. Clinical and radiological follow-up

A single senior neurosurgeon, who routinely follows iCCF patients treated with GKRS at our institution, performed all clinical and radiological assessments to ensure consistency in evaluation criteria and follow-up procedures. After the procedure, clinical outcomes were evaluated by 1 neurosurgeon at 2 weeks, 1 month, 3 months, 6 months, and 12 months (Fig. 1D–G).

Radiological follow-up was performed using brain MRI and MRA at 3, 6, and 12 months after treatment.

### 2.4. Statistical analysis

Statistical analysis was performed using Statistical Package for the Social Sciences version 23.0 (IBM Corp., Armonk). Continuous variables are summarized as mean ± standard deviation or median (interquartile range), and categorical variables as counts (percentages). Time-to-event outcomes were defined as: time to symptomatic improvement, measured from the date of GKRS to the 1st documented resolution of the presenting symptom complex (ocular pain, chemosis, proptosis, or intraocular pressure [IOP] elevation); and time to radiologic obliteration, measured from GKRS to the 1st MRI/MRA showing complete disappearance of the fistulous flow signal in the cavernous sinus. Patients without the event by the last follow-up were right-censored at that visit. Kaplan–Meier estimates were generated for both outcomes.

## 3. Results

The patient demographics, clinical characteristics, and treatment parameters of the study cohort are summarized in Table 1. The mean follow-up period was 47.4 months (range, 12–108 months). The mean age of the 20 patients was 64.2 years (range, 46–86 years), with 18 females and 2 males. The mean interval from symptom onset to treatment was 1.8 months (range, 1–4 months). Clinical presentations included conjunctival and eyelid edema in 19, elevated IOP in 16 (mean IOP, 27.7 mm Hg; range, 19–42 mm Hg), chemosis in 10, ocular pain in 9, proptosis in 9, headache in 8, and diplopia in 7 patients.

**Table 1:** Patient demographics, clinical features, and treatment parameters.

Parameter	Value
Number of patients	20
Age, mean ± SD (range), years	64.2 ± 13 (46–86)
Sex, n (%)	
Female	18 (90%)
Male	2 (10%)
Follow-up period, mean ± SD (range), months	47.4 ± 25 (12–108)
Interval from symptom onset to treatment, mean ± SD (range), months	1.8 ± 0.9 (1–4)
Clinical presentations, n (%)	
Conjunctival and eyelid edema	19 (95%)
Elevated intraocular pressure (IOP)	16 (80%)
Chemosis	10 (50%)
Ocular pain	9 (45%)
Proptosis	9 (45%)
Headache	8 (40%)
Diplopia	7 (35%)
Mean IOP, mm Hg (range)	27.7 (19–42)
Barrow classification, n (%)	
Type B	6 (30%)
Type C	10 (50%)
Type D	4 (20%)
Venous drainage involvement, n (%)	
Superior ophthalmic vein (SOV) dilation	16 (80%)
Sphenoparietal sinus (SPS) dilation	3 (15%)
Both SOV and SPS dilation	1 (5%)
Mean SOV diameter (range), mm	3.59 (1.6–7.29)
Mean SPS diameter (range), mm	2.91 (2.6–3.3)
Mean target volume (range), cm^3^	0.53 (0.13–1.64)
Mean margin dose (range), Gy	17.65 (16–18)
Mean number of isocenters (range)	8.5 (2–26)
Mean conformity index (range), %	97 (93–100)
Mean time to complete radiologic obliteration, mean ± SD (range), months	5.3 ± 2.8 (1–12)

IOP = intraocular pressure, SD = standard deviation, SOV = superior ophthalmic vein, SPS = sphenoparietal sinus.

According to the Barrow classification, indirect CCF subtypes were type B in 6 patients, type C in 10, and type D in 4. Four patients with contralateral symptoms were treated at the primary fistula site in 2 patients and the symptom site in 2 patients. The draining venous systems involved the superior ophthalmic vein (SOV) and the sphenoparietal sinus (SPS). The SOV dilatation was observed in 16 patients, the SPS dilatation in 3, and both in 1 patient. The mean SOV diameter was 3.59 mm (range, 1.6–7.29 mm), and the mean SPS diameter was 2.91 mm (range, 2.6–3.3 mm). Of the 4 patients with expanded SPS, 2 were Barrow type C, 1 was B, and 1 was D.

**Figure 2.**
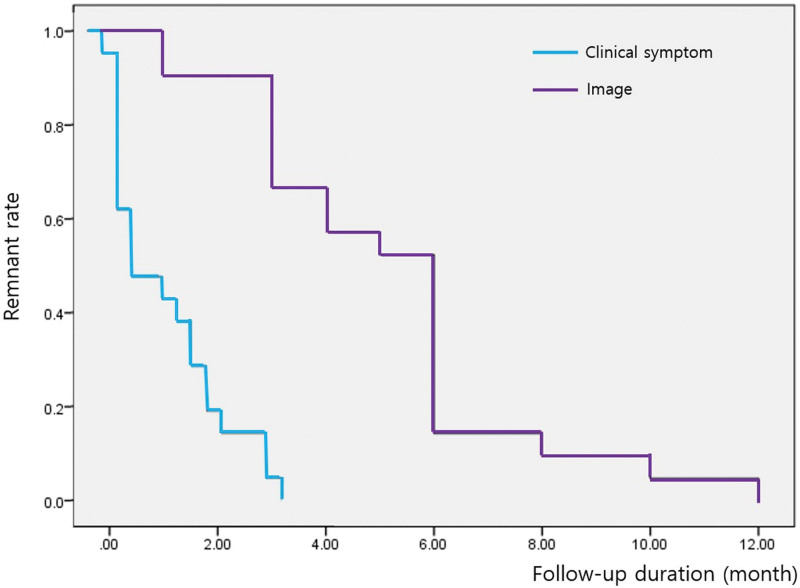
Kaplan–Meier curves showing the rate of remnant fistula according to follow-up duration after GKRS. The blue line represents time to clinical symptom improvement, and the purple line represents time to radiologic obliteration. Tick marks indicate censored observations. Numbers at risk are shown below each x-axis.

The mean target volume was 0.53 cm^3^ (range, 0.13–1.64 cm^3^). The mean margin dose was 17.65 Gy (range, 16–18 Gy). The mean number of isocenters was 8.5 (range, 2–26), and the mean conformity index was 97% (range, 93%–100%). The mean time to clinical symptom relief was 1.3 months (range, 0.25–3.25 months). The mean time to complete radiologic obliteration was 5.3 months (range, 1–12 months, Fig. 2). The overall obliteration rate was 100%, and there were no complications.

## 4. Discussion

In the present study, standalone GKRS for iCCFs demonstrated excellent clinical and radiologic outcomes. All 20 patients achieved complete obliteration (100% obliteration rate) without any treatment-related complications. Clinical symptoms improved rapidly, with a mean time to symptom relief of 1.3 months, and complete radiologic obliteration was achieved at a mean of 5.3 months. The treatment was well tolerated in all patients, including those of advanced age, and no adverse events were observed during a mean follow-up period of 47.4 months.

Taken together with prior series reporting 58% to 100% obliteration after radiosurgery for iCCF, our 100% obliteration without complications suggests that carefully planned standalone GKRS may serve as a definitive option for selected nonemergent iCCF, particularly in elderly or medically fragile patients.

Carotid-cavernous fistulas (CCFs) have historically been managed with surgical techniques and, more recently, with endovascular treatment (EVT), particularly transvenous embolization using coils, which has become the current gold standard due to its capacity for immediate symptom relief.^[13]^ However, EVT is not without limitations: it requires vascular access, carries procedural risks such as vessel injury or cranial nerve palsy, and may not be feasible in patients with poor general condition or challenging venous anatomy.

So, less invasive treatment strategies such as SRS and particularly GKRS have emerged as alternative modalities.^[14,15]^ Although initially applied to patients who failed EVT or were unfit for surgical intervention, accumulating evidence suggests that indirect CCFs are more radiosensitive and demonstrate higher obliteration rates than other types of dural arteriovenous fistulas when treated with GKRS. Furthermore, GKRS offers a noninvasive, outpatient-based therapeutic option with a favorable safety profile, making it particularly attractive in elderly or medically fragile patients.^[16,17]^ Given these advantages, systematic investigation of GKRS as a definitive treatment modality for indirect CCFs is of significant clinical importance. Clarifying its efficacy, safety, and long-term outcomes could broaden the therapeutic landscape, potentially establishing GKRS not merely as a salvage option but as a primary alternative or complementary strategy alongside EVT.

### 4.1. Clinical outcome

In our study, complete obliteration of indirect CCF at 12-month follow-up was 100%. In 1991, Barcia-Salorio et al addressed an initial experience of radiosurgery for low-flow CCF, and reported a 91% cure rate in low-flow fistula.^[18]^ Later, several studies showed favorable outcomes of SRS for CCF, with a 58.5% to 100% complete obliteration rate.^[9,17,19–21]^ In 2024, Maroufi et al reported that the obliteration rate of CCF with stereotactic radiosurgery alone was 94.4%, procedure-related complication 8.1%.^[11]^ Based on 7 published case series, the treatment of CCF with SRS has shown a complete obliteration rate of these lesions that ranges from 50% to 100%.^[18,19,21–25]^ The authors cited the advantages of using SRS when the vascular lesion was inaccessible endovascularly or when EVT would have resulted in a high risk of complications.^[21–25]^ Two large studies of 206 and 146 patients preferred radiosurgery.^[14,15]^ Obliteration rates were significantly lower (70% in one of these studies and 73% in the other) compared to the overall obliteration rate in the EVT group (83%). In a systematic review of SRS of intracranial dural fistulas, complete obliteration of iCCFs was achieved in 73% of the cases.^[13]^ However, Voldřich et al reported that the clinical outcome in the radiosurgery group was slightly better than in EVT (98% vs 91% clinical improvement rate).^[26]^ The results suggest radiosurgery to be an equally viable treatment option, especially in patients with non-acute clinical symptomatology and without cortical venous drainage. We show our results with a previous report on the outcome of GKRS monotherapy for CCF in Table 2. These findings are consistent with prior reports demonstrating high obliteration rates after radiosurgery and further support standalone GKRS as an effective and safe treatment option for carefully selected iCCF patients.

**Table 2:** Literature review of standalone GKRS for CCF.

Authors (yr)	No. of patients	Mean follow-up (mo, range)	Volume (cm^3^)	Irradiation dose (Gy), isodose line (%)	Complete obliteration (latest angiography)	Procedure-related complications
Barcia-Salorio et al^[19]^	22	49.8 (15–172)	–	30–40	20/22 (90.9%)	0
Guo et al^[23]^	18	12 (6–27)	–	28 (22–38)	12/18 (67%)	0
Jung et al^[9]^	5	5 (3–6)	–	–	5/5 (100%)	0
Park et al^[20]^	18	30 (6–65)	2.6 (0.6–11.6)	17 (14–19)	15/18 (83%)	0
Hung et al^[17]^	20	31 (13.5–48.5)	–	22, 50%	17/20 (85%)	5/20 (25%)
Wu et al^[14]^	123	4–213	1.4–8.8	15.5–18.9 (23.2–27.9)	72/123 (58.5%)	NA
Kranawetter et al^[12]^	17	>3 yr	0.6 (0.1–2.4)	18 (18–20), 50%–75%	12/14 (86%)	0
Present study	20	47.4 (12–108)	0.53 (0.13–1.64)	16–18, 50%–60%	20/20 (100%)	0

NA = not available.

In our patient group, 4 patients presented with ocular symptoms contralateral to the fistula site. Two patients underwent treatment on the ipsilateral side of the fistula, and 2 patients on the contralateral side. All 4 patients experienced complete resolution of the fistula and symptoms. Similar cases have been reported in other papers.^[27–29]^ One patient in our group showed bilateral symptoms, had bilateral fistula, and we targeted both fistulas. Although not present in our patient’s case, bilateral ocular symptoms may occur due to a unilateral fistula. In that bilateral manifestation, there is a high probability that the fistula is draining into cortical veins.^[28]^

### 4.2. Duration of symptomatic and radiologic improvement

The mean time to clinical symptom relief was 1.3 months (range, 0.25–3.25 months). The mean time to complete radiologic obliteration was 5.3 months (range, 1–12 months). Other reports have shown that symptom relief typically begins 2 to 3 months after radiosurgery, and MRI shows fistula regression after 6 to 9 months.^[30]^ When analyzing the symptom improvement period according to target volume, there was no relationship between volume and symptom improvement or speed of obliteration on the image (*P*-value > .217, *P*-value > .618). The mechanism of fistula site occlusion is the same as that of arteriovenous malformation occlusion, which is due to intimal hypertrophy induced by irradiation. However, it is thought that the occlusion of the CCF is induced more promptly due to the difference in compact irradiation due to the smaller target volume compared to the arteriovenous malformation. In our series, the mean target volume was 0.53 cm^3^ (range, 0.13–1.64 cm^3^). Taken together, the rapid clinical and radiologic responses observed in our cohort align well with previous studies and highlight the favorable biological behavior and radiosensitivity of iCCFs treated with GKRS.

### 4.3. Draining venous system-related clinical symptom and outcome differences

In our patients, the SOV dilatation was observed in 16 patients, the SPS dilatation in 3, and both in 1 patient. The patients draining through the SOV presented with ocular symptoms and elevated IOP, while those draining through the SPS had minimal ocular symptoms and complained of headache. However, no differences in treatment outcomes were observed. Given the small number of patients, further analysis with a larger patient population is needed.

Miller reported that iCCFs that drain anteriorly usually produce visual symptoms and signs.^[31]^ In the mildest cases, there is redness of 1 or, rarely, both eyes caused by dilation and arterialization of both conjunctival and episcleral veins.^[31]^ In these cases, the appearance may suggest a primary ocular disorder, such as conjunctivitis, episcleritis, or thyroid eye disease; however, a careful examination of the dilated vessels usually demonstrates a typical tortuous corkscrew appearance that is virtually pathognomonic of an iCCF.^[28,31]^ There also may be minimal eyelid swelling, conjunctival chemosis, proptosis, or a combination of these findings.^[31]^

When iCCFs drain posteriorly into the superior and inferior petrosal sinuses, they are usually asymptomatic. In most of these cases, there is no evidence of orbital congestion.^[2,28]^ In most cases of ocular motor nerve paresis caused by a posteriorly draining iCCF, the onset of the paresis is sudden, and only one of the ocular motor nerves is affected. The oculomotor nerve is most often affected, and the resulting paresis may be complete with the involvement of the pupil, incomplete with pupil involvement, or incomplete with pupil sparing. In almost all cases, the paresis is associated with ipsilateral orbital or ocular pain, a presentation that initially suggests an intracranial aneurysm.^[32]^ The correct diagnosis in such cases is not evident until cerebral angiography is performed. In other cases, the posteriorly draining fistula produces an abducens or trochlear nerve paresis, again usually associated with ocular or orbital pain.^[32]^ Dural fistulas that drain posteriorly sometimes cause brainstem congestion that may be associated with neurologic deficits. In addition, such fistulas may produce intracranial hemorrhage.^[33]^

We examined the IOP and drainage pathways in relation to the patients’ ocular discomfort, and found that IOP was higher when draining to the SOV. Elevated IOP is thought to be a symptom of secondary glaucoma, and its mechanism is that raised episcleral venous pressure may produce increased IOP.^[29]^ Angle-closure glaucoma develops from elevated orbital venous pressure, congestion of the iris and choroid, and onward displacement of the iris-lens diaphragm.^[34]^ Improvement in clinical symptoms related to discomfort in the eye itself began before improvement in the photograph, but improvement in increased IOP was about 3 months later than other symptoms, and it is thought that time is needed for the changed structures to recover anatomically. Although symptom patterns varied according to venous drainage pathways, treatment outcomes remained uniformly favorable across all groups, supporting the reliability of GKRS regardless of drainage pattern.

### 4.4. Considerable complications and advantages of GKRS

In our series, no complications or delayed adverse events were observed. The radiation dose to the optic apparatus remained <3 Gy, a level widely considered safe for the optic pathways. In radiosurgery, the steep dose gradient and precise imaging contribute to sparing the optic structures and brainstem from high-dose irradiation.^[24]^

Compared with the optic nerve, other cranial nerves within the cavernous sinus are generally more resistant to radiation-related injury, with reports indicating tolerance up to 40 Gy in a single fraction.^[35]^ In our patients, the maximum dose to the cavernous sinus wall was 20 Gy – well below the 40 Gy threshold – and no new cranial nerve palsy was detected during follow-up. Careful treatment planning is essential in minimizing radiosurgical complications.^[36]^

Among our patients, elevated IOP normalized after GKRS. In CCF, elevated IOP primarily results from increased episcleral and vortex vein pressures. Closure of the fistula and restoration of venous circulation typically lead to IOP reduction. In some cases, however, glaucoma may arise from iris neovascularization due to reduced retinal perfusion, or from vascular engorgement and edema of the choroid and ciliary body. These mechanisms can produce anterior displacement of the iris/lens complex and pupil-block glaucoma.^[37]^ When glaucoma develops as a complication of SRS, doses to the choroid and ciliary body often exceed 13 to 20 Gy.^[38]^ In our study, these structures received <0.5 Gy, which likely explains the absence of glaucoma following GKRS.

Previous reports have noted carotid artery stenosis after GKRS for cavernous meningiomas and pituitary adenomas.^[39]^ In such cases, the carotid artery may receive 25 to 30 Gy, and occasionally up to 40 Gy, a range associated with increased risk of stenosis.^[40,41]^ Some studies also suggest that heterogeneous dose distribution with hot spots directly involving the carotid artery further increases the risk.^[41]^ In our study, the carotid artery received <16 Gy, a level not considered detrimental for stenosis development.

Dry eye syndrome has been linked to median doses of ≥4 Gy to the lacrimal gland.^[42]^ In our study, the dose was only 0.5 Gy, likely accounting for the absence of dry eye symptoms in our patients.

Radiation-induced cataract formation is a common late effect, with latency periods ranging from 6 to 64 months (typically 2–3 years).^[43]^ The generally accepted threshold for cataractogenesis is around 2 Gy to the lens, though some evidence suggests even 0.5 Gy may be harmful.^[44]^ Consequently, both GKRS and cerebral angiography have been associated with higher rates of cataract formation compared with controls.^[45]^ In our series, however, the lens dose was 0.0 Gy, and no cataracts developed during follow-up exceeding 3 years.

We think the absence of complications and the rapid achievement of occlusion were attributable to meticulous treatment planning. Specifically, only a 4-mm collimator was used, conformity was optimized at 97% (range, 93%–100%), and multiple isocenters (mean 8.5; range, 2–26) were employed to ensure homogeneous radiation within the target while minimizing exposure to surrounding tissues. These dosimetric advantages, together with the absence of radiation-related complications in our series, reinforce GKRS as a safe modality when meticulous planning is performed.

### 4.5. Limitations and points requiring further research

Although our results showed an excellent obliteration rate with little radiation adverse effects after properly adjusted delivery of radiation to vulnerable structures, the small number of patients is the biggest limitation of this study. A larger patient population and more detailed analysis are needed in the future. Also, although the use of a single neurosurgeon helped maintain consistency in clinical and radiological assessments, it may also introduce the possibility of observer bias. Going 1 step further, it will be necessary to confirm the safety and efficacy of treatment in patients with cortical reflux, and it will also be necessary to analyze whether there are differences in results according to differences in the number of isocenters and conformity when planning treatment with other institutions.

## 5. Conclusion

Even with a small number of patients in this study, indirect CCF treated with GKRS likely affords a high obliteration rate without complications. Standalone GKRS can be an option in selected indirect CCF patients without seriously increased IOP and without aggressive venous cortical reflux.

## Author contributions

**Methodology:** Jung-Soo Park.

**Data curation:** Eun Jeong Koh.

**Formal analysis:** Eun Jeong Koh.

**Investigation:** Eun Jeong Koh.

**Project administration:** Eun Jeong Koh.

**Visualization:** Eun Jeong Koh.

**Writing – original draft:** Eun Jeong Koh.

**Writing – review & editing:** Jung-Soo Park.

## References

[1] Barrow DL Spector RH Braun IF Landman JA Tindall SC Tindall GT. Classification and treatment of spontaneous carotid-cavernous sinus fistulas. J Neurosurg. 1985;62:248–56. 3968564 10.3171/jns.1985.62.2.0248

[2] Miller NR. Carotid-cavernous fistulas. In: Miller NR Newman NJ Biousse V Kerrison JB, eds. Walsh & Hoyt’s Clinical Neuro‑Ophthalmology. 6th ed. Lippincott Williams & Wilkins; 2005:2263–96.

[3] Ellis JA Goldstein H Connolly ES Jr Meyers PM. Carotid-cavernous fistulas. Neurosurg Focus. 2012;32:E9. 10.3171/2012.2.FOCUS1223 22537135

[4] Travers B. A case of aneurism by anastomosis in the orbit, cured by the ligature of the common carotid artery. Med Chir Trans. 1811;2:1–420. 10.1177/095952871100200101 PMC2128834 20895126

[5] Gandhi D Chen J Pearl M Huang J Gemmete JJ Kathuria S. Intracranial dural arteriovenous fistulas: classification, imaging findings, and treatment. AJNR Am J Neuroradiol. 2012;33:1007–13. 22241393 10.3174/ajnr.A2798 PMC8013238

[6] Miller TR Gandhi D. Intracranial dural arteriovenous fistulae: clinical presentation and management strategies. Stroke. 2015;46:2017–25. 25999384 10.1161/STROKEAHA.115.008228

[7] Barcia-Salorio JL Hernández G Broseta J González‑Darder J Ciudad J. Radiosurgical treatment of a carotid‑cavernous fistula. In: Szikla G, ed. Stereotactic Cerebral Irradiation. Inserm Symposium No. 12. Holland Biomedical Press; 1979:251–6.

[8] Desai R Rich KM. Therapeutic role of gamma knife stereotactic radiosurgery in neuro-oncology. Mo Med. 2020;117:33–8. 32158047 PMC7023953

[9] Jung HH Chang JH Whang KM Pyen JS Chang JW Park YG. Gamma Knife surgery for low-flow cavernous sinus dural arteriovenous fistulas. J Neurosurg. 2010;113:21–7. 21121783 10.3171/2010.8.GKS10977

[10] Kim MJ Hong SW Kim DJ. Efficacy and safety of stereotactic radiosurgery versus endovascular treatment for symptomatic cavernous sinus dural arteriovenous fistula without ophthalmological emergency: a single-center 10-year experience. J Neurosurg. 2023;139:139–49. 36461825 10.3171/2022.10.JNS221770

[11] Maroufi SF Fallahi MS Ghasemi M Sheehan JP. Stereotactic radiosurgery with versus without embolization for intracranial dural arteriovenous fistulas: a systematic review and meta-analysis. Neurosurg Focus. 2024;56:E6. 10.3171/2023.12.FOCUS23797 38427988

[12] Kranawetter B Cho A Hirschmann D. Radiosurgery as a stand-alone treatment option for cerebral dural arteriovenous fistulas: the Vienna series. J Neurol Surg A Cent Eur Neurosurg. 2025;86:48–55. 38151030 10.1055/a-2235-5256

[13] Alexander MD Halbach VV Hallam DK. Long-term outcomes of endovascular treatment of indirect carotid cavernous fistulae: superior efficacy, safety, and durability of transvenous coiling over other techniques. Neurosurgery. 2019;85:E94–E100. 30418600 10.1093/neuros/nyy486

[14] Wu HM Pan DH Chung WY. Gamma Knife surgery for the management of intracranial dural arteriovenous fistulas. J Neurosurg. 2006;105:43–51. 18503329 10.3171/sup.2006.105.7.43

[15] Chen CJ Lee CC Ding D. Stereotactic radiosurgery for intracranial dural arteriovenous fistulas: a systematic review. J Neurosurg. 2015;122:353–62. 25479123 10.3171/2014.10.JNS14871

[16] Pan DHC Wu HM Kuo YH. Intracranial dural arteriovenous fistulas: natural history and rationale for treatment with stereotactic radiosurgery. Prog Neurol Surg. 2014;27:176–94. 10.1159/000341793 23258522

[17] Hung YC Mohammed N Kearns KN. Stereotactic radiosurgery for cavernous sinus versus noncavernous sinus dural arteriovenous fistulas: outcomes and outcome predictors. Neurosurgery. 2020;86:676–84. 31384943 10.1093/neuros/nyz260 PMC7317986

[18] Barcia-Salorio JL Soler F Barcia JA Hernández G. Radiosurgery of carotid‑cavernous fistulae. Acta Neurochir Suppl. 1994;62:10–2. 7717123 10.1007/978-3-7091-9371-6_3

[19] Barcia-Salorio JL Soler F Barcia JA Hernández G. Stereotactic radiosurgery for the treatment of low‑flow carotid‑cavernous fistulae: results in a series of 25 cases. Stereotact Funct Neurosurg. 1994;63:266–70. 7624645 10.1159/000100330

[20] Park SH Park KS Kang DH Hwang JH Hwang SK. Stereotactic radiosurgery for dural carotid cavernous sinus fistulas. World Neurosurg. 2017;106:836–43. 28465265 10.1016/j.wneu.2017.04.143

[21] Wu CA Yang HC Hu YS. Venous outflow restriction as a predictor of cavernous sinus dural arteriovenous fistula obliteration after Gamma Knife surgery. J Neurosurg. 2019;132:132–9. 30684940 10.3171/2018.9.JNS182040

[22] Söderman M Edner G Ericson K. Gamma Knife surgery for dural arteriovenous shunts: 25 years of experience. J Neurosurg. 2006;104:867–75. 16776329 10.3171/jns.2006.104.6.867

[23] Guo WY Pan DH Wu HM. Radiosurgery as a treatment alternative for dural arteriovenous fistulas of the cavernous sinus. AJNR Am J Neuroradiol. 1998;19:1081–7. 9672015 PMC8338652

[24] Koebbe CJ Singhal D Sheehan J. Radiosurgery for dural arteriovenous fistulas. Surg Neurol. 2005;64:392–8; discussion 398. 16253680 10.1016/j.surneu.2004.12.026

[25] Pollock BE Nichols DA Garrity JA Gorman DA Stafford SL. Stereotactic radiosurgery and particulate embolization for cavernous sinus dural arteriovenous fistulae. Neurosurgery. 1999;45:459–66; discussion 466. 10493367 10.1097/00006123-199909000-00008

[26] Voldřich R Charvát F Beneš V Netuka D. What is the most effective method to treat indirect carotid‑cavernous fistula? Neurosurg Rev. 2022;46:9. 36482213 10.1007/s10143-022-01923-z

[27] Stiebel‑Kalish H Setton A Berenstein A. Bilateral orbital signs predict cortical venous drainage in cavernous sinus dural AVMs. Neurology. 2002;58:1521–4. 12034790 10.1212/wnl.58.10.1521

[28] Miller NR. Diagnosis and management of dural carotid–cavernous sinus fistulas. Neurosurg Focus. 2007;23:E13. 10.3171/FOC-07/11/E13 18004961

[29] Yu SCH Cheng HKM Wong GKC Chan CM Cheung JYL Poon WS. Transvenous embolization of dural carotid‑cavernous fistulae with transfacial catheterization through the superior ophthalmic vein. Neurosurgery. 2007;60:1032–7; discussion 1037. 17538376 10.1227/01.NEU.0000255455.05355.31

[30] Kim DJ Kim DI Suh SH. Results of transvenous embolization of cavernous dural arteriovenous fistula: a single‑center experience with emphasis on complications and management. AJNR Am J Neuroradiol. 2006;27:2078–82. 17110671 PMC7977219

[31] Miller NR. Dural carotid–cavernous fistulas: epidemiology, clinical presentation, and management. Neurosurg Clin N Am. 2012;23:179–92. 22107868 10.1016/j.nec.2011.09.008

[32] Lin HL Hu TT. Isolated third nerve palsy with pupillary involvement resulting from carotid-cavernous sinus fistula: a case report. Medicine (Baltim). 2019;98:e14472. 10.1097/MD.0000000000014472 PMC6380736 30732214

[33] Harding AE Kendall B Leonard TJK Johnson MH. Intracerebral haemorrhage complicating dural arteriovenous fistula: a report of two cases. J Neurol Neurosurg Psychiatry. 1984;47:905–11. 6481384 10.1136/jnnp.47.9.905 PMC1027989

[34] Bujak M Margolin E Thompson A Trobe JD. Spontaneous resolution of two dural carotid‑cavernous fistulas presenting with optic neuropathy and marked congestive ophthalmopathy. J Neuroophthalmol. 2010;30:222–7. 20498622 10.1097/WNO.0b013e3181ceb483

[35] Mehta MP Kinsella TJ. Cavernous sinus cranial neuropathies: is there a dose‑response relationship following radiosurgery? Int J Radiat Oncol Biol Phys. 1993;27:477–80. 8407425 10.1016/0360-3016(93)90262-t

[36] Shen CC Tsuei YS Yang MY. Gamma Knife radiosurgery for indirect dural carotid–cavernous fistula: long‑term ophthalmological outcome. Life (Basel). 2022;12:1175. 36013354 10.3390/life12081175 PMC9410130

[37] Cruciani F Lorenzatti M Nazzarro V Abdolrahimzadeh S. Bilateral acute angle closure glaucoma and myopia induced by topiramate. Clin Ter. 2009;160:215–6. 19756324

[38] Gigliotti CR Modorati G Di Nicola M. Predictors of radio‑induced visual impairment after radiosurgery for uveal melanoma. Br J Ophthalmol. 2018;102:833–9. 28903963 10.1136/bjophthalmol-2017-310801

[39] Ito H Onodera H Sase T. Percutaneous transluminal angioplasty in a patient with internal carotid artery stenosis following Gamma Knife radiosurgery for recurrent pituitary adenoma. Surg Neurol Int. 2015;6:S279–83. 26069850 10.4103/2152-7806.157795 PMC4450501

[40] Shin M Kurita H Sasaki T. Stereotactic radiosurgery for pituitary adenoma invading the cavernous sinus. J Neurosurg. 2000;93:2–5. 11143249 10.3171/jns.2000.93.supplement

[41] Spatola G Frosio L Losa M Del Vecchio A Piloni M Mortini P. Asymptomatic internal carotid artery occlusion after Gamma Knife radiosurgery for pituitary adenoma: report of two cases and review of the literature. Rep Pract Oncol Radiother. 2016;21:555–9. 27721669 10.1016/j.rpor.2016.09.006 PMC5045960

[42] Horwath‑Winter J Schneider MR Wackernagel W. Influence of single‑fraction Gamma‑Knife radiosurgery on ocular surface and tear function in choroidal melanoma patients. Br J Ophthalmol. 2013;97:466–70. 23349246 10.1136/bjophthalmol-2012-302402

[43] Kennerdell JS Flores NE Hartsock RJ. Low‑dose radiotherapy for lymphoid lesions of the orbit and ocular adnexa. Ophthalmic Plast Reconstr Surg. 1999;15:129–33. 10189642 10.1097/00002341-199903000-00012

[44] Stewart FA Akleyev AV Hauer‑Jensen M. ICRP publication 118: ICRP statement on tissue reactions and early and late effects of radiation in normal tissues and organs—threshold doses for tissue reactions in a radiation protection context. Ann ICRP. 2012;41:1–322. 10.1016/j.icrp.2012.02.001 22925378

[45] Liang CL Liliang PC Chen TB. The risk of cataractogenesis after Gamma Knife radiosurgery: a nationwide population‑based case‑control study. BMC Ophthalmol. 2017;17:40. 28376826 10.1186/s12886-017-0435-1 PMC5381080

